# Supplementary material for: Structural basis for ELL2 and AFF4 activation of HIV-1 proviral transcription
Source: Nat Commun. 2017 Jan 30;8:14076. doi: 10.1038/ncomms14076 (PMC5290273; doi:10.1038/ncomms14076)
Supplement: Supplementary Information — Supplementary figures and Supplementary Tables. [file ncomms14076-s1.pdf]

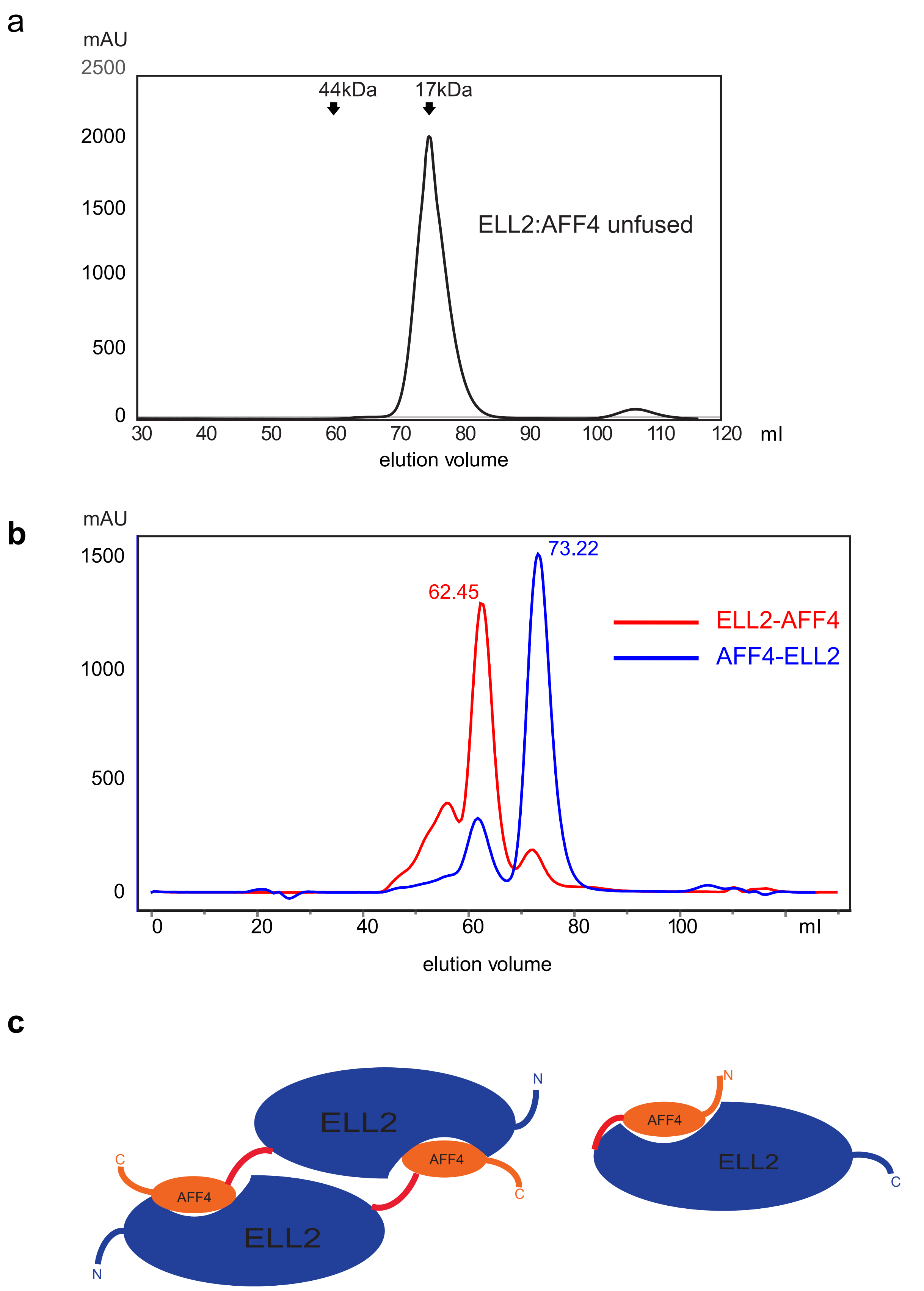

Supplementary Figure 1. a. The unfused ELL2Occ:AFF4ELLBow complex is monomeric in solution. b. The ELL2Occ–(Gly-Ser)4- AFF4ELLBow was eluted at 62.45ml on the Hiload 16/60(GE) while AFF4ELLBow–(Gly-Ser)4-ELL2Occ was eluted at 73.22 ml, which correspond to a dimer and a monomer, respectively. Red line: ELL2Occ–(Gly-Ser)4-AFF4ELLBow , Blue line: AFF4ELLBow–(Gly-Ser)4-ELL2Occ . c. Schematic of hypothesis for ELL2Occ–(Gly-Ser)4-AFF4ELLBow dimerization in solution while AFF4ELLBow–(Gly-Ser)4-ELL2Occ was monomeric. ELL2Occ is shown in blue. AFF4ELLBow is shown in orange. N, C represent amino termini and carboxyl termini respectively.

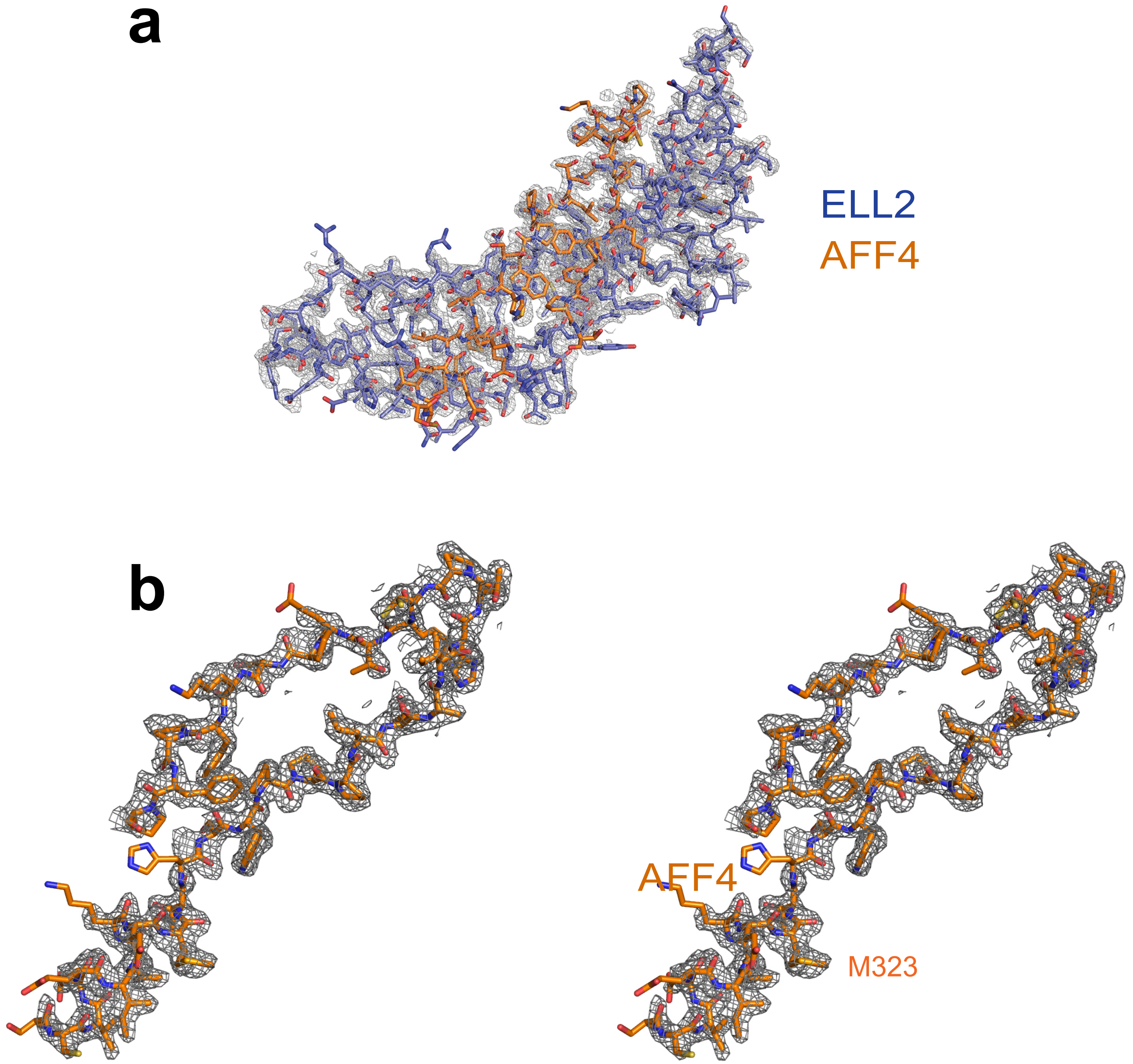

Supplementary Figure 2. a. The overall experimental electron density map after density modification is displayed at a contour level of 2σ (gray), with AFF4ELLBow and ELL2Occ shown as stick in orange and light blue, respectively. b. Stereo view of the portion of the map from (A) corresponding to AFF4ELLBow, displayed at a contour level of 2σ (gray), with AFF4ELLBow shown in a stick model. Met323 is highlighted.

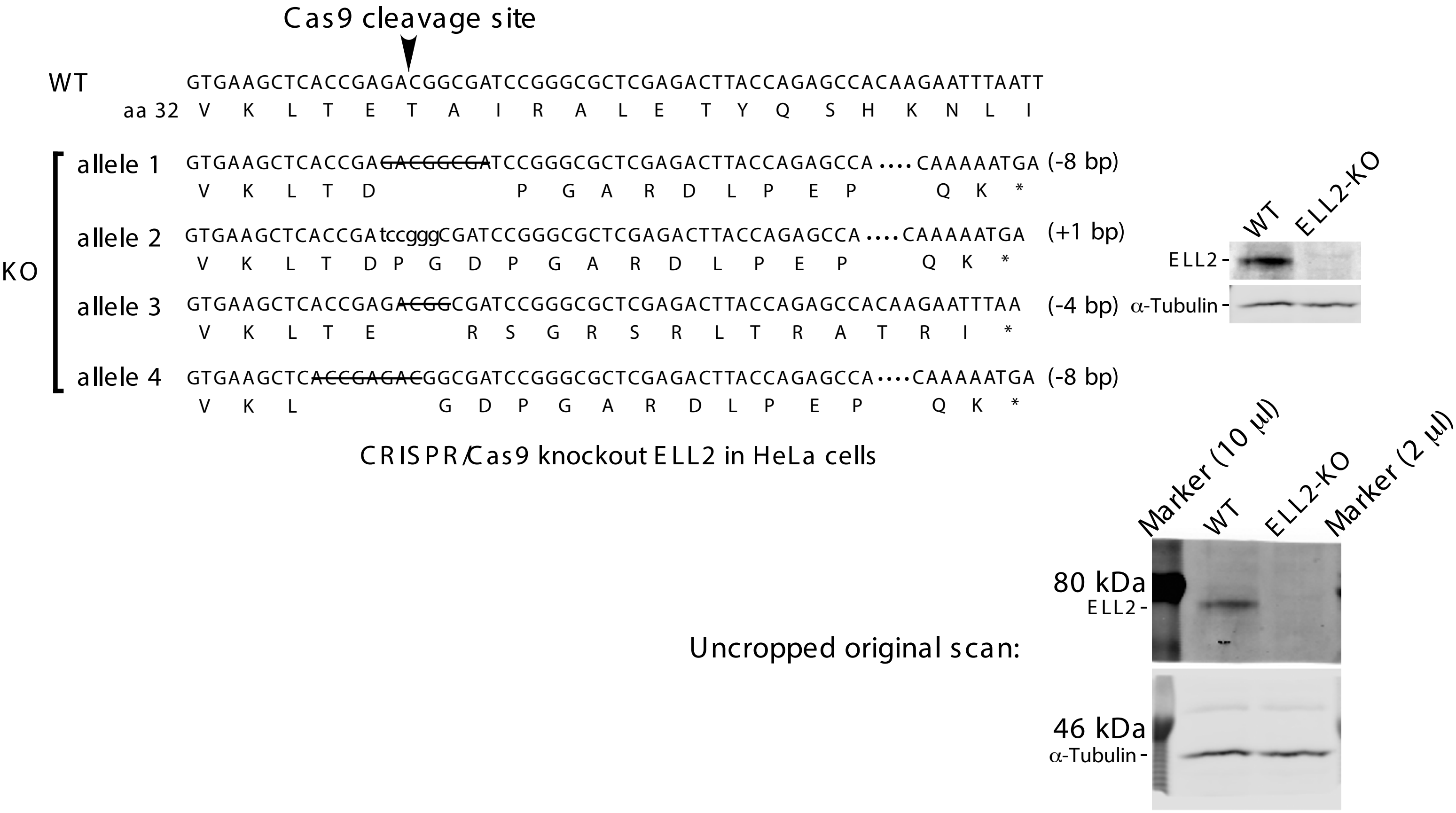

Supplementary Figure 3. Verification of disruption of the ELL2 gene by CRISPR-Cas9 in the HeLa-derived ELL2 knockout cell line ΔELL2. DNA and predicted amino acid sequences surrounding the intended Cas9 cleavage site (arrowhead) in the wild-type ELL2 gene and its mutant alleles generated by CRISPR-Cas9 are shown. Insertions of extra nucleotides are indicated by lowercase letters, deletions are indicated by capital letters containing strike-throughs, and the omitted nucleotides are marked by four consecutive dots. Premature stop codons as a result of frame-shift mutations are indicated by stars. The loss of ELL2 protein expression in ΔELL2 was confirmed by immunoblotting with the indicated antibodies.

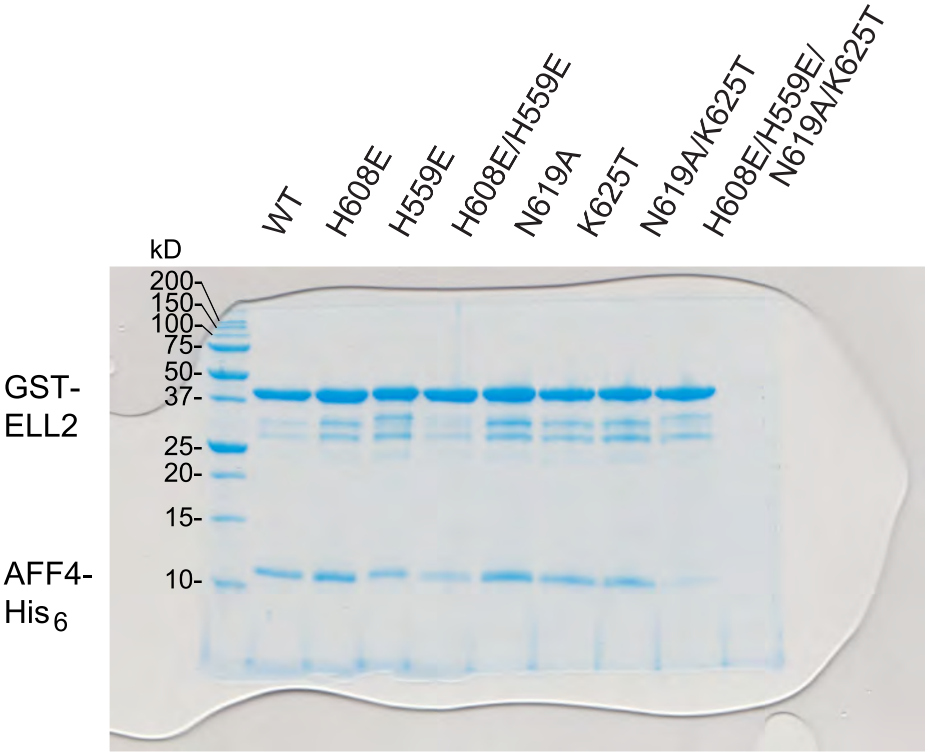

Supplementary Figure 4. Uncropped version of gel shown in Figure 5c.

A

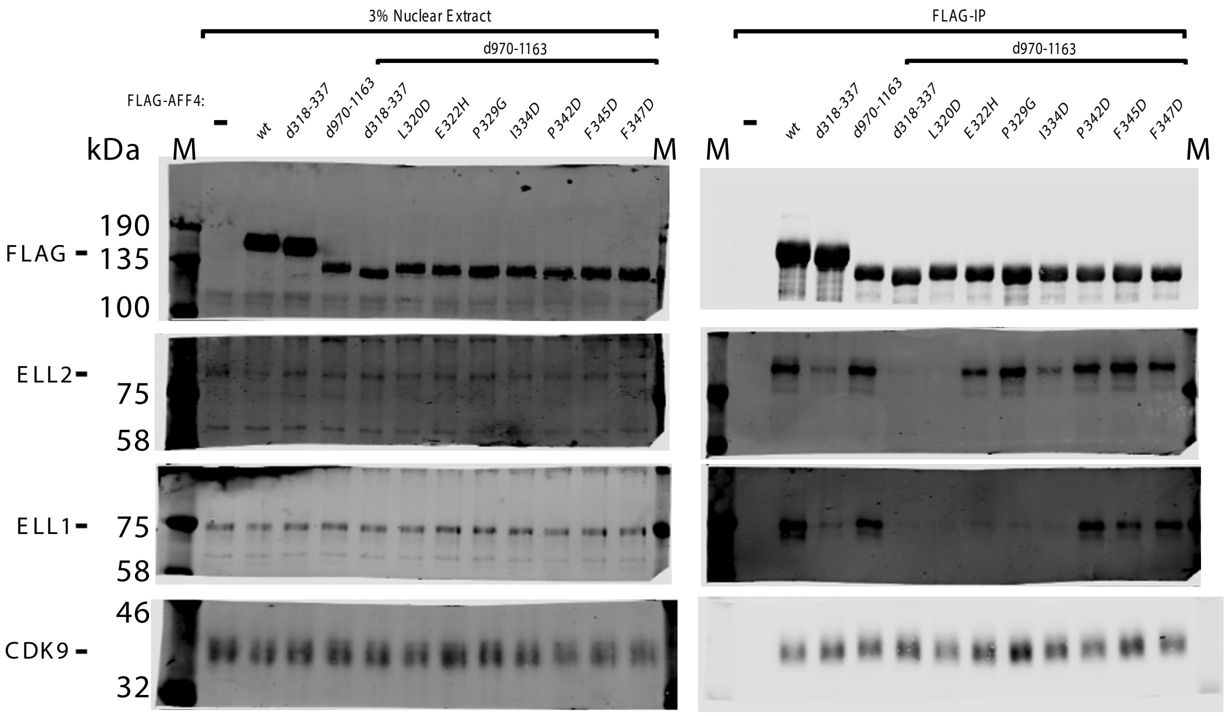

B

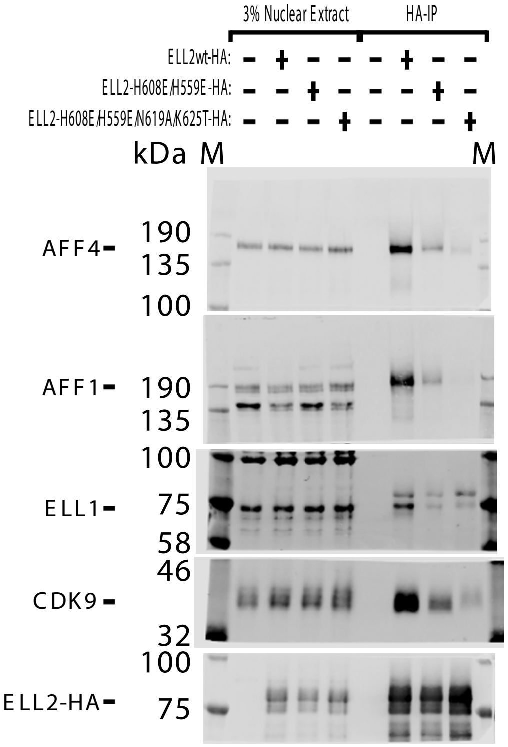

Supplementary Figure 5. Uncropped versions of the gels shown in Figure 6.

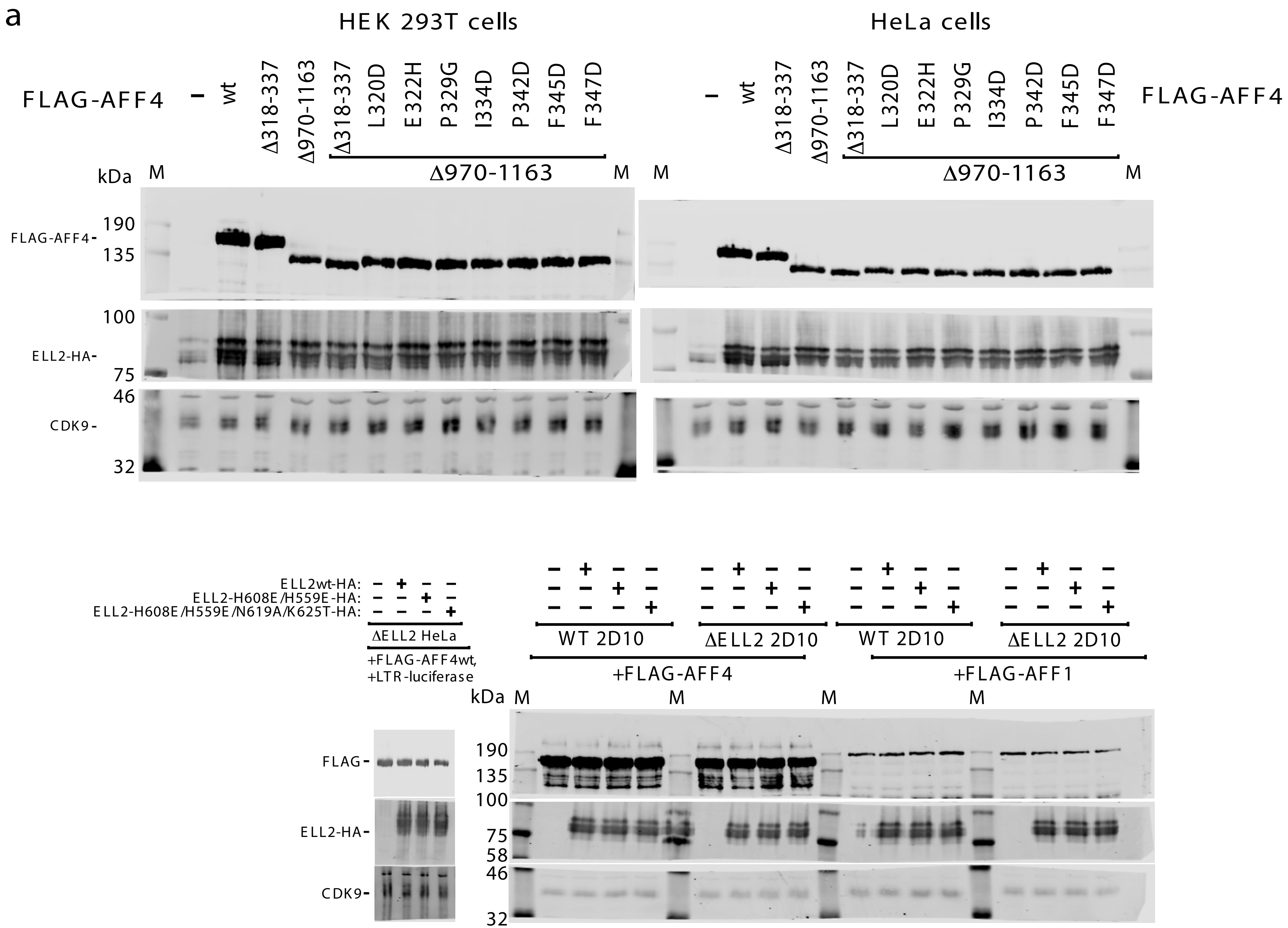

Supplementary Figure 6. Uncropped versions of the gels shown in Figure 7.

Supplementary Table 1. K_d values for AFF4-ELL2 binding as determined by fluorescence anisotropy. Fluorescence anisotropy data in Figure 5a, b were fitted with a single site binding model. NA means fitting was not applicable because of very low affinity binding.

**AFF4 construct/mutation**	**K_d (μM)**	**R^2**
WT, 300-350 +His$_6$-tag	0.086 $\pm$ 0.024	0.97
WT, 318-350 peptide	4.0 + 1.57	0.91
V316D	0.23 + 0.048	0.99
I319D/L320D	5.4 + 1.87	0.92
M323D	2.7 + 1.14	0.88
D317P/E318P	0.80 + 0.18	0.99
K321P	0.24 + 0.06	0.98
E322H	0.13 + 0.033	0.99
W327D	0.37 + 0.086	0.96
P328G/P329G	0.27 + 0.098	0.97
L331D	1.6 + 0.31	0.98
T332K	0.069 + 0.037	0.97
I334D	0.67 + 0.21	0.95
L331D/I334D	1.9 + 0.51	0.97
P342D	0.2 + 0.05	0.99
F345D/F347D	0.13 + 0.058	0.97
I319D/L232D/M323D	NA	
D317P/E318P/E322H	NA	
M323D/L331D/I334D	NA	
W327D/ L331D/I334D	NA	

Supplementary Table 2. Primers used for PCR and mutagenesis in this study.

PCR for Recombinant expression		
**Gene**	**Forward Primer**	**Reverse Primer**
ELL2(519-640)	GTTGTTGGATCCGAACCTTCAGCAATTGAAC	CCTTCACTCGAGTTATTGCTGTTGGTCAAATTC
AFF4(300-350)	GTTGTTGGATCCCCTTCCCAACCACTG	CCTTCACTCGAGTTACTCCTTAGTTGGAAAAG
ELL2(519-640)-4GS-AFF4(300-350)	CAAGCAGAGTCATGGTCCggtagtggtagtggtagtggtagtCCTTCCCAACCACTG	CAGTGGTTGGGAAGGactaccactaccactaccactaccGGACCATGACTCTGCTTG
AFF4(300-350)-4GS-ELL2(519-640)	CTTTTCCAACTAAGGAGggtagtggtagtggtagtggtagtGAACCTTCAGCAATTG	CAATTGCTGAAGGTTCactaccactaccactaccactaccCTCCTTAGTTGGAAAAG
**mutations**		
**ELL2**		
V593D	CATGAAGAAGaCTTACAAGAATATC	TTCTTGTAAGtCTTCTTCATGAAC
H608E	CCCAATTACgaaGAAGAAAAATAC	TTTTTCTTCttcGTAATTGGGACTAG
F548E	AAGGATGACgaCAATGCAGAGTATG	CTCTGCATTGtcGTCATCCTTATAATTC
Y555E	TATGATGAGgaCAGAGCTTTGCATG	CAAAGCTCTGtcCTCATCATACTC
H559E	AGAGCTTTGgaaGCCAGGATGGAG	CATCCTGGCttcCAAAGCTCTGTAC
D632A	GAATTTGcaCAACAGCAAGCAG	CTGTTGtgCAAATTCACCTATTAG
N619A	CTTCATgcCAAGCTGGCTCACATC	CAGCTTGgcATGAAGATATTCACATC
K625T	CACATCacAAGGATGATAGGTG	CATCCTTgtGATGTGAGCCAGCTTG
**AFF4**		
V316D	TGTGAGCTGTGatGATGAAATC	TTTCATCatCACAGCTCACATC
I319/320D	ATGAAgaCgatAAAGAGATGAC	TCTTTatcGtcTTCATCCACACAG
M323D	CTAAAAGAGgatACGCATTCATG	TGAATGCGTatcCTCTTTTAGGATTTC
317D318E	AGCTGTGTGccTccAATCCTAAAAGAGATG	TTTTAGGATTggAggCACACAGCTCAC
K321P	GAAATCCTAccAGAGATGACGCATTC	CGTCATCTCTggTAGGATTTCATC
E322H	ATCCTAAAAcacATGACGCATTCATG	ATGCGTCATgtgTTTTAGGATTTC
W327D	ACGCATTCAgatCCTCCCCCTCTAAC	AGGGGGAGGatcTGAATGCGTCATCTC
L331D	CCTCCCCCTgatACGGCTATTCATAC	AATAGCCGTatcAGGGGGAGGCCATG
D317A	TGTGTGGcTGAAATCCTAAAAC	GATTTCAgCCACACAGCTCACATC
T332K	CCTCTAAaGGCTATTCATACACCATG	AATAGCCtTTAGAGGGGGAGGCCATG
I334D	CTAACGGCTgaTCATACACCATG	TGGTGTATGAtcAGCCGTTAGAG
P342D	AAAACAGAAgaTTCCAAATTTC	AAATTTGGAAtcTTCTGTTTTGCATG
P3282930G	CATTCATGGggTggCggTCTAACGGCTATTCTCATGGggTggCggTCTAACGGCTATTC	AGCCGTTAGAccGccAccCCATGAATGCGTCCGTTAGAccGccAccCCATGAATGCGTCATC
F345347D	CCTTCCAAAgaTCCTgaTCCAACTAAGGAG	CTTAGTTGGAtcAGGAtcTTTGGAAGGTTC
V316D/I319/320D/M323D/317D318E	CTGTGatGATGAAgacgatAAAGAGgatACGCATTCATGGTGGATGAAgaCgatAAAGAGgatACGCATTCATGGCCTC	TGAATGCGTatcCTCTTTatcgtcTTCATCatCACAGCTCACATCATGCGTatcCTCTTTatcGtcTTCATCCACACAGCTCAC
L331D/I334D	CCCTgatACGGCTgatCATACACCATG	GTATGatcAGCCGTatcAGGGGGAGGCCATG
**PCR for *In Vivo*Assay**		
**Gene**	**Forward Primer**	**Reverse Primer**
AFF4-d318-337	GCTTCTGGTGATGTGAGCTGTGTGGATTGCAAAACAGAACCTTCCAAATTTCCTTTTCC	GGTTCTGTTTTGCAATCCACACAGCTC
AFF4-d970-1163	GAATCCAAATCCCCATTCCCTATGtgaTCGAGCATGCATCTAGAGGGC	CTCGATCACATAGGGAATGGGG
AFF4-L320D	ATGTGAGCTGTGTGGATGAAATCGACAAAGAGATGACGCATTCATGGC	TCATCTCTTTGTCGATTTCATCCAC
AFF4-E322H	AGCTGTGTGGATGAAATCCTAAAACACATGACGCATTCATGGCCTCCC	TGCGTCATGTGTTTTAGGATTTCATC
AFF4-P329G	AGAGATGACGCATTCATGGCCTGGTCCTCTAACGGCTATTCATACACC	CGTTAGAGGACCAGGCCATGAATG
AFF4-I334D	GGCCTCCCCCTCTAACGGCTGACCATACACCATGCAAAACAGAACC	ATGGTGTATGGTCAGCCGTTAGAG
AFF4-P342D	GCTATTCATACACCATGCAAAACAGAAGACTCCAAATTTCCTTTTCCAACTAAGG	AAAGGAAATTTGGAGTCTTCTGTTTTGC
AFF4-F345D	CCATGCAAAACAGAACCTTCCAAAGACCCTTTTCCAACTAAGGAGTCTCA	GTTGGAAAAGGGTCTTTGGAAGGTT
AFF4-F347D	CAAAACAGAACCTTCCAAATTTCCTGACCCAACTAAGGAGTCTCAGCAGT	TCCTTAGTTGGGTCAGGAAATTTGG
ELL2-N619A/K625T	GAAAAATACAGATGTGAATATCTTCATgcaAAGCTGGCTCACATCaccAGGCTAATAGGTGAATTTGACCAAC	GTGATGTGAGCCAGCTTTGCATG
ELL2-H559E	GAGTATGATGAGTACAGAGCTTTGgagGCCAGGATGGAGACTGTAGCTAG	CATCCTGGCCTCCAAAGCTCTG
ELL2-H608E	GATAAAGCAGTCTAGTCCCAATTACgagGAAGAAAAATACAGATGTGAATATCTTC	GTATTTTTCTTCCTCGTAATTGGGACTAG
